# On Exudation

**Published:** 1847-08

**Authors:** John Hughes Bennett

**Affiliations:** Lecturer on Pathology, and the Practice of Physic; Director of the Poly-Clinic, at the Royal Dispensary, Edinburgh, &c.


*On Exudation.—Its Development.*—By John Hughes Bennett, M. D., F. R. S. E., Lecturer on Pathology, and the Practice of Physic; Director of the Poly-Clinic, at the Royal Dispensary, Edinburgh, &c.

*Enclysted Growths.*—The different crypts and follicles of the skin, and mucous membrane, as well as several of the excretory ducts of internal organs may become obstructed, and as a consequence enlarged and hypertrophied. It is true such growths usually consist of one or more elementary tissues, and should not on this account be constituted a class of themselves. Their importance in a practical point of view, however, as well perhaps as the difficulty of knowing where to describe such compound growths, warrant our speaking of them under a separate head.

Encysted growths are composed of a cyst or membranous envelope, enclosing various kinds of contents. They differ greatly in size, situation and structure, which renders their arrangement somewhat difficult. By some they have been divided into *simple* and *compound*, according as the tumour is formed of one cyst, or is composed of several. By others they have been arranged according to the nature of their contents into *hygromatous*, or aqueous; *atheromatous*, resembling gruel; *melicerous*, honey-like, and *steatomatous*, or fatty encysted growths. The latter mode of division is very faulty, the atheromatous, melicerous, and steatomatous varieties being all more or less fatty, whilst some kinds of compound encysted tumours contain different contents in different cysts. But as there can be no doubt that the peculiar contents give to these growths a distinctive character, we shall first speak of them as simple or compound, and then describe their different kinds of contents.

*Simple encysted growths.*—These growths are formed of a cyst composed of fibrous tissue, lined by a smooth membrane. Sometimes the membrane is structureless, or only composed of areolar tissue. At other times it is lined with a distinct layer of epithelial cells, the nuclei of which are very apparent on the addition of acetic acid. The former kind constitutes the vesicles so frequently found in the plexus choroides, kidneys, ovaries, &c., varying in size from a pin's head to that of a hazel nut, or even walnut, usually with aqueous contents. The latter kind constitute the cystic growths arising in the follicles of the skin, in the mamma, ovaries, testicles, &c., which frequently reach the size of an orange, are sometimes much larger, and vary greatly as to the nature of their contents. For the most part they are only sparingly supplied with blood-vessels, and seldom cause inconvenience except from the deformity they occasion when situated externally.

*Compound encysted growths* are of two kinds. The external sac may contain on its internal surface secondary or even tertiary cysts, which may be sensile or pedunculated—or the growth may be divided into numerous departments by divisions of the fibrous sac. This is the true multilocular encysted tumour. The external cyst in all these cases is formed of fibrous tissue. The internal surface is smooth, sometimes with, at others without an epithelial layer. The primary as well as the enclosed cysts are for the most part richly supplied with blood-vessels, and hence they are peculiarly prone to contain exudation which has undergone various kinds of development, as well as to ulcerate. These growths frequently attain an enormous size measuring several feet in circumference, whilst their internal membrane may secrete more or less rapidly gallons of fluid.

The *contents* of encysted growths are very various, and give, as we have previously stated, a peculiar character to them.

1. The contents may be a perfectly colourless fluid, resembling water, or the limpid serum so frequently secreted in the lateral ventricles of the brain. It is structureless, and chemically contains a minute proportion of salts, and a certain amount of albumen which coagulates on boiling. Such are frequently the contents of so-called serous cysts, or false hydatids of the plexus choroides, kidneys, ovaries, &c. A *hydrocele*, and other dropsies of shut serous sacs, may be looked on pathologically as constituting a form of hygromatous encysted growth.

2. The contained fluid may have an amber or golden yellow colour, and resemble the serum formed after the coagulation of the blood. It is still structureless, but contains a large amount of albumen, as is proved by the action of heat and nitric acid.

3. The contents are more or less gelatinous, sometimes slightly so, like weak gelatine—at others firm, capable of being cut with a knife like tolerably strong glue or firm calves-foot jelly. The colour of the gelatinous matter may vary from a slight yellowish tinge, to a deep amber, or brownish yellow colour. Sometimes this matter is structureless, at others it may be seen to contain very delicate filaments, combined with pale oval bodies, the outlines of which become stronger on the addition of acetic acid. This re-agent frequently causes the gelatinous mass to coagulate into a firm white fibrous structure, capable of being separated by needles, and presenting all the structure of filamentous tissue. This kind of contents is common in the ovary, and we have seen it in the kidney and other organs. On one occasion the gelatinous matter in the kidney, contained numerous granules, and more than once we have found in the centre of clear amber masses of it a creamy white substance, either wholly granular, or in the process of formation into pus corpuscles.

4. The cyst may be distended with epithelial scales, which have evidently been thrown off from its internal surface, and become compressed together, and partially broken down. Hence on examination, clusters of such scales may be found mixed with numerous debris, and fat granules and globules, sometimes with crystals of cholesterine. The contents of the cysts are usually of a white or slightly yellow colour, which is sometimes fluid, at others semi-solid. The *molluscum contagiosum* of dermatologists is thus constituted. The small cystic swelling for the most part originate in the crypts of the skin, which are more or less enlarged.

5. The contents may consist principally of fat, either amorphous, crystallized, or organized, that is cellular. If amorphous they resemble honey, constituting the *melicerous* growths of morbid anatomists. In many cases, however, where the yellow colour is uniform, when it breaks down under the finger and closely resembles honey to the naked eye, faint cell walls more or less compressed together may be observed by the microscope.

At other times the fatty contents are of a whitish colour, occurring in masses of a pearly aspect and smooth surface, mingled with a roughened yellowish, and more granular fatty matter. This white matter consists of numerous crystals of cholesterine placed in a close juxta-position,—the granular fatty matter of oil globules and granules, mixed with broken up crystals, epithelial scales, and sometimes the products of fibrinous exudation. Such is the general structure of the *atheromatous* encysted growths of various authors.

Again the fatty matter may be more or less lardaceous in character, and consists of beautiful round or oval cells, some of which are distinctly nucleated. Mixed with these may be a granular matter, combined with epithelial cells or their debris. At other times no distinct cells can be observed, only a granular or amorphous mass, the most part of which is soluble in ether. This constitutes the *steatomatous* encysted growth.

6. Many encysted growths contain hair and teeth. The hair is occasionally inserted into the walls of the cyst, at others exists loose, mixed with the fatty or other contents. They are exactly of the same structure as the hairs in other parts of the body, having distinct bulbous roots. When attached they are surrounded by a follicle in the lining membrane, when loose they have been evidently grown in follicles, and afterwards become separated. Their apices are frequently split up into several fibres in the longitudinal direction. The *teeth* belong sometimes to the first, and sometimes to the second dentition. They present on section the usual structure of cavity, with ivory, enamel, and bone. Sometimes they are found embedded in a follicle of the lining membrane, at others like the hair quite loose.

7. Occasionally the cysts contain lymph, softened fibrine, and purulent matter, composed of plastic, pus, and compound granular cells, the result of exudation into their cavities. Occasionally the serous fluid is more or less mixed up with extravasation of blood, giving to the contained liquid various colours and appearances, according to the period the extravasation has taken place. Thus it may be red, dark brown resembling coffee, of a dark greenish tinge, &c., &c. Sometimes it is of a dark bluish or blackish tint from excess of pigmentary deposit.

8. Sometimes the contents of the cystic growth are formed of a solid exudation, which has undergone the sarcomatous transformation as previously described, and wholly consists of fusiform cells. The exudation poured into such cysts may pass into the cancerous formation, when the characters we have described will be associated with those yet to be detailed which distinguish cancer.

9. Some cysts contain the peculiar secretion of the organ in which they are found. The cysts in the liver are full of bile, and those in the kidney of urine.

The mode in which encysted growths are developed is generally by the hypertrophy of pre-existing tissues, whereby canals are distended, follicles or vesicles enlarged, and their walls thickened. Thus the simple cysts in the plexus choroides are owing to effusion of serum into the areolar spaces of the villi of the membrane, and the subsequent distension of the membrane. Those in the kidney are owing to the distension of uriniferous tubes above an accidental obstruction, in the same manner that the whole kidney may become encysted from obstruction of the ureter. In like way the crypts of the skin or follicles of mucous membranes become obstructed at their orifice, and their contents accumulating, gradually distend the walls, which become enlarged and thickened. Simple cysts in the ovary become dilated by enlargement of the Graafian vesicles, either deep in the stroma of the organ, or on the surface, when they grow outwards and become pedunculated.

The origin of compound encysted tumours is not so well determined. It is very probable, however, that in most cases they consist of clusters of simple cysts, which become compressed together, and are at length surrounded by a capsule. They are most common in the ovary; and here we can readily understand how successive growths of Graafian vesicles may give rise either to the appearance of secondary or tertiary cysts, or to the multilocular form we have described. In all cases as the compound cyst enlarges, the internal ones open into each other by ulceration. Hence, in very old compound cysts we find one large cavity, with the traces on its internal wall of previously existing cysts, or bands and divisions, with pouches between them, indicating where previous cysts has existed.

Another mode in which compound cysts are formed is by the gradual enlargement of the areolæ in newly formed fibrous tissue. On examining thin sections of sarcomatous growths, we observe the filamentous tissue arranged in a circular form, enclosing spaces varying in size from the $\frac{1}{50}$ of a millemetre, to several inches in diameter. When of a certain size they are often lined by a distinct epithelial membrane, and many contain serum, blood, or exudation, either in a granular or fibrous state. Such growths have long been known under the name of *cystic sarcoma.*

The diagnosis of encysted growths belongs to the *special* pathology of each organ affected by them, and will constitute the subject of a distinct communication. It need only be mentioned here that a knowledge of the structure of these tumours is not unimportant, as an examination of the fluids discharged from them frequently enables us to speak with certainty regarding their nature.

An acquaintance with the structure and mode of development of these growths must convince us that there are only two modes of *treatment* applicable—namely, 1st, entire extirpation, and 2d, destruction of the secreting surface in their interior. The idea that a dense fibrous envelope, often containing numerous secondary cysts, all richly furnished with blood-vessels can be absorbed through the agency of mercury, iodine, or any other drug, must be purely imaginary. Neither can it be supposed, that as long as any of the cysts remain intact, a cure can be hoped for. But we have seen that the natural course of these secondary cysts is to open into each other, until at length only one large cyst remains. Under such circumstances a rupture, by exciting adhesive exudation, and thus destroying the secreting surfaces, or inducing adhesions between them may cause a radical cure. It is in this manner that the occasional spontaneous removal of certain ovarian cysts are to be explained. In one such case, we had an opportunity of examining under the care of Dr. Makellar, the walls of the compound cyst were found after death shrunk and thickened, and the whole growth in process of obliteration.—*Monthly Journ. of Med. Sci.*

